# Draft Genome Sequence of Streptomyces sp. Strain DH-12, a Soilborne Isolate from the Thar Desert with Broad-Spectrum Antibacterial Activity

**DOI:** 10.1128/genomeA.00108-18

**Published:** 2018-03-01

**Authors:** Junjing Jiao, Julia Paterson, Tobias Busche, Christian Rückert, Jörn Kalinowski, Dharmesh Harwani, Harald Gross

**Affiliations:** a Department of Pharmaceutical Biology, Institute of Pharmaceutical Sciences, University of Tübingen, Tübingen, Germany; b German Centre for Infection Research (DZIF), Partner Site Tübingen, Tübingen, Germany; c Technology Platform Genomics, CeBiTec, Bielefeld University, Bielefeld, Germany; d Department of Microbiology, Maharaja Ganga Singh University, Bikaner, Rajasthan, India

## Abstract

Strain DH-12 exhibits broad-spectrum antibacterial activity toward Gram-positive and Gram-negative pathogens. The 7.6-Mb draft genome sequence gives insight into the complete secondary metabolite production capacity and reveals genes putatively responsible for its antibacterial activity, as well as genes which enable the survival of the organism in an extreme arid environment.

## GENOME ANNOUNCEMENT

The unique chemical scaffolds and huge metabolic potential of streptomycetes have made them the most promising candidates for bioprospecting studies (1). The likelihood of isolating undiscovered streptomycetes from regular terrestrial habitats has greatly diminished; therefore, unusual habitats, such as deserts, are being explored for the discovery of species producing novel metabolites (2, 3). The large number of biosynthetic gene clusters in Streptomyces genomes has strengthened the belief in their potential to facilitate the discovery of yet-to-be-identified secondary metabolites in the near future (4–6). The Thar Desert in India represents an arid desert ecosystem (7) which is underexplored in terms of microbial biodiversity. During a screening program for antimicrobial compounds from the thermophilic microbes native to the Thar Desert, Streptomyces strain DH-12 was isolated from a soil sample collected in the Bikaner region (28°01ʹN, 73°22ʹE) falling in the India-administered area of the Thar Desert. This work presents the annotated genome of strain DH-12 and analyses uncovering its biosynthetic capacity for secondary metabolism.

The genome of DH-12 was sequenced using a combined Illumina/PacBio sequencing approach. Genomic DNA (gDNA) was first subjected to paired-end sequencing with an Illumina HiSeq platform. The first *de novo* assembly was performed utilizing Newbler version 2.8 and yielded 440 contigs. To improve the quality of the sequence, the genome was resequenced using the PacBio RS II technology (10-kb library, 150,567 reads, 1,119 Mb, and 7,438 bp average read length). The quality of the Illumina reads was improved by trimming off low-quality bases using BBDuk (BBMap suite version 36.77). High-quality reads were assembled into contigs using ABySS version 2.0.2 (8). The long reads were mapped to the draft assembly using BLASR (9). Subsequently, the contigs were linked and placed into scaffolds. The orientation, order, and distance between the contigs were estimated using SSPACE-LongRead version 1.0 (10). Using Illumina reads, gapped regions within scaffolds were (partially) closed using GapFiller version 1.10 (11). Finally, assembly errors and nucleotide disagreements between the Illumina reads and scaffold sequences were corrected using Pilon version 1.21 (12). The final draft genome assembly resulted in 13 contigs totaling 7,604,612 bp, with 147.3-fold overall coverage and a G+C content of 72.8%. The closest related type strains based on the 16S rRNA gene sequence are Streptomyces xylophagus NBRC 13845 (GenBank accession number AB184526), Streptomyces variabilis NRRL B-3984 (NR_043840), and Streptomyces werraensis NBRC 13404 (NR_112390), all with 97% sequence identity.

Automated secondary metabolism analysis using antiSMASH 4.0.2 (13) predicted 19 biosynthetic gene clusters (BGCs). Eight of these matched, at a high level of similarity (89 to 100%), known clusters for an alkylresorcinol (14), antimycin (15), albaflavenone (16), actinomycin (17), desferrioxamine B (18), and hopene (19) and twice for ectoine (20). The production of actinomycins D, I, and V was experimentally proven employing tandem mass spectrometry (MS/MS) and high-resolution MS (HR-MS) analyses of the corresponding isolated pure compounds. The duplicated ectoine BGCs contribute to survivability under extreme conditions. The remaining clusters were predicted to encode 2 terpenes, 1 siderophore, 2 polyketide synthases (PKSs), 1 nonribosomal peptide synthetase (NRPS), and 5 hybrid-NRPS-PKSs.

### Accession number(s).

This whole-genome sequencing (WGS) project and the partial 16S rRNA sequence have been deposited at DDBJ/ENA/GenBank under the accession numbers PPFB00000000 and KM205638, respectively.
